# No association between hepatitis C virus infection and risk of colorectal cancer: a systematic review and meta-analysis of cohort studies

**DOI:** 10.3389/fmed.2024.1327809

**Published:** 2024-06-04

**Authors:** Cheng Chang, Hong-Mei Yan, Yan-Lin Liao

**Affiliations:** ^1^Department of Gastroenterology, Wuhan Hospital of Traditional Chinese Medicine, Wuhan, China; ^2^Department of Cardiovascular Medicine, Wuhan Hospital of Traditional Chinese Medicine, Wuhan, China

**Keywords:** colorectal cancer, cohort studies, systematic review, meta-analysis, hepatitis C virus

## Abstract

**Background and aim:**

There is still uncertainty regarding whether hepatitis C virus (HCV) infection is associated with colorectal cancer (CRC). This study aims to investigate the potential association between HCV infection and CRC through a systematic review and meta-analysis of cohort studies.

**Methods:**

PubMed, Embase, and Web of Science were systematically searched from the beginning of their inception to October 2023 to find relevant cohort studies on the association between HCV infection and CRC risk. The random-effect, generic inverse variance method was used to calculate the hazard ratios (HRs) and 95% confidence intervals (CIs) for CRC outcome among individuals with HCV infection. We also performed subgroup and sensitivity analysis.

**Results:**

A total of 8 cohort studies involving 1,939,164 participants were included in this meta-analysis. The result from the meta-analysis suggested that there was no statistically significant association between HCV and the risk of developing CRC (HR = 0.99, 95% CI: 0.82–1.88, *p* = 0.88) with low statistical heterogeneity (I^2^ = 28%, *p* = 0.20). Subgroup analyses that were conducted based on study design, diagnosis of HCV infection, and publication year yielded similar results. Analyses of subgroups based on study areas revealed that there was no significant association between HCV infection and CRC risk in Asia (*n* = 2, HR = 0.96, 95% CI: 0.71–1.29, *p* = 0.79; I^2^ = 26%), Europe (*n* = 3, HR = 1.06, 95% CI: 0.83–1.37, *p* = 0.63; I^2^ = 0%), and North America (*n* = 2, HR = 1.10, 95% CI: 0.87–1.38, *p* = 0.44; I^2^ = 0%); however, a negative correlation was found in Oceania (*n* = 1, HR = 0.43, 95% CI: 0.22–0.84, *p* = 0.01). Sensitivity analysis further reinforce the stability of our conclusion.

**Conclusion:**

Our cohort-based meta-analysis showed insufficient evidence to support the association between HCV infection and an increased risk of CRC. To gain a clearer insight into the potential association between these two conditions, it would be beneficial to conduct large, well-designed, high-quality prospective cohort studies that consider different ethnic populations and potential confounding factors.

**Systematic review registration**: PROSPERO, identifier [CRD42023472688], https://www.crd.york.ac.uk/prospero/display_record.php?ID=CRD42023472688.

## 1 Introduction

Colorectal cancer (CRC) is the third most common cancer worldwide and the second leading cause of death. In 2020, it was estimated that there would be 1.9 million new cases of CRC, including those of the anus, and 935,000 deaths, accounting for 10% of all cancer cases and deaths (1). Notably, incidence rates increased significantly among younger people (aged under 50) during the period from 1990 to 2019, especially in countries with a high socio-demographic Index (2). By 2030, the proportion of young-onset CRC is expected to be 11% for colon cancer and 23% for rectal cancer (3). Current treatments for CRC patients include endoscopic and surgical local resection, preoperative radiotherapy and systemic treatment, metastatic local ablation therapy, palliative chemotherapy, targeted therapy, and immunotherapy (4). Although these new treatments have increased the overall survival time for late-stage diseases to 3 years (4), they are not able to provide the ideal treatment. Since symptoms of this disease only manifest in the advanced stages, it is essential for public health to implement CRC prevention and screening, explore the risk factors of CRC, prevent CRC according to these risk factors, and improve the early detection rate of cancer. It has been established that colorectal cancer is caused by a combination of genetic and environmental risk factors (4).

Hepatitis C virus (HCV), a single stranded RNA virus in the flaviviridae family, is a liver-affecting virus that can cause both acute and chronic hepatitis (5). Around the world in 2020, an estimated 57 million people had chronic HCV infection, the prevalence of HCV RNA viraemic being 0.7%. The vast majority of these individuals were located in low- and middle-income countries (6). The top five countries with the highest HCV burden are China, India, Pakistan, Russia, and the USA, and 80% of the HCV-infected population is from 30 countries (6). Long-term HCV infection can cause permanent harm to the liver, resulting in cirrhosis, a decline in liver health, and hepatocellular carcinoma (5, 7). Apart from the liver, HCV is able to infect other cells as well, resulting in a variety of extrahepatic cancers, such as non-Hodgkin lymphoma, pancreatic cancer, and cholangiocarcinoma (8–11).

Recently, the relationship between HCV infection and CRC has received widespread attention. A previous meta-analysis of five studies (two cohort and three case–control studies) indicated that patients with HCV infection had a significantly greater risk of CRC than those without HCV infection (12). In their meta-analysis, however, only a few cohort and case–control studies were included. Furthermore, the previous meta-analysis did not consider newly published cohort studies and partially overlooked cohort studies. Cohort studies are the most effective and reliable method to establish causal relationships in non-interfering relationships. We therefore conducted a systematic review and meta-analysis of cohort studies to gain a better understanding of the potential association between HCV infection and CRC risk. This will aid in the development of more effective prevention strategies for CRC.

## 2 Materials and methods

Registration of this study protocol has already been completed on the PROSPERO platform (registration number: CRD42023472688). This study was conducted in line with the PRISMA 2020 reporting criteria for Systematic Reviews and Meta-Analysis.

### 2.1 Data sources and search strategy

From the beginning of their inception to October 12, 2023, searches of the PubMed, Embase, and Web of Science databases were conducted without any language limitation. The main search terms included “hepatitis C,” “HCV,” “colon,” “rectum,” “colorectal,” “colons,” “colonic,” “rectal,” “cancer,” “cancers,” “tumour,” “tumor,” “tumours,” “tumors,” “neoplasms,” “neoplasm,” “neoplasia,” and “carcinoma.” The search strategy incorporated both medical subject headings (MeSH) and free words. The full search strategy is available in Supplementary Table S1. To find additional studies, we manually checked the reference lists of cohort studies and other published meta-analyses.

### 2.2 Study selection criteria

In order to be eligible for inclusion, studies had to meet the following criteria: (1) cohort studies (prospective or retrospective) that examined the link between HCV infection and the risk of CRC; (2) exposure to HCV infection; (3) outcome of the incidence rate of CRC; (4) presentation of relative risks (RRs), hazard ratios (HRs), standardized incidence ratios (SIRs), standardized morbidity ratios (SMRs), or data for their calculation, with corresponding 95% confidence intervals (CIs). When multiple studies are conducted on the same cohort or overlapping populations, the study with the largest sample size will be the one taken into account. We excluded case–control or cross-sectional studies, duplicate publications, conference abstracts, editorials, comments, animal studies, reviews, and meta-analyses, as well as studies with insufficient data. Two reviewers assessed all potential studies to confirm they met the above inclusion and exclusion criteria. Any disputes were settled through mutual agreement.

### 2.3 Data extraction and quality assessment

The two reviewers extracted the same data: the first author’s surname, year of publication, study design, geographic region, sample size, mean age, confirmation method for HCV and CRC, follow-up duration, HRs/RRs/SIRs/SMRs and their 95% CIs, and adjusted confounders. Any discrepancies were decided by a third researcher.

The Newcastle-Ottawa Scale (NOS) was used to evaluate the methodological quality of the studies included (13). This scale evaluates a study according to three criteria, with a maximum of four stars for the selection of participants, two stars for the comparability of the study groups, and three stars for the determination of outcomes of interest, giving a total of nine stars. We rate studies of nine stars as high quality, seven or eight stars as medium quality, and six stars or less as low quality (14).

### 2.4 Statistical analysis

All statistical analyses were conducted using the Review Manager 5.3 (The Cochrane Collaboration, Copenhagen, Denmark) and STATA/SE 12.0 (STATA Corporation, Texas, United States). To measure the effect size of each eligible study, pooled HRs and 95% CIs were used. Studies that reported HRs with varying degrees of covariate adjustment were examined to identify the HRs that best adjusted for potential confounders. A random effects meta-analysis, based on the DerSimonian and Laird method (15), was conducted to calculate the pooled adjusted HRs and 95% CI of all eligible studies. As the outcome of interest was uncommon, SMRs, RRs, and SIRs were approximately equal to HRs (16). The Cochran’s *Q*-test (*p* ≤ 0.10) and I^2^ statistic were used to evaluate the statistical heterogeneity. I^2^ values of 0–25% indicate insignificant heterogeneity, while 26–50% demonstrate low heterogeneity, 51–75% signify moderate heterogeneity, and 76–100% represent high heterogeneity (17). Subgroup analyses were conducted in order to evaluate any potential factors that could influence the overall results and to recognize potential sources of heterogeneity. To determine the stability of the results, a sensitivity analysis was performed by removing each of the included studies one at a time. Assessing the potential publication bias, the funnel plots were inspected and Begg’s and Egger’s tests (18, 19) were performed. Statistical significance was determined by a *p*-value of less than 0.05.

## 3 Results

### 3.1 Literature selection process

**Figure 1:**
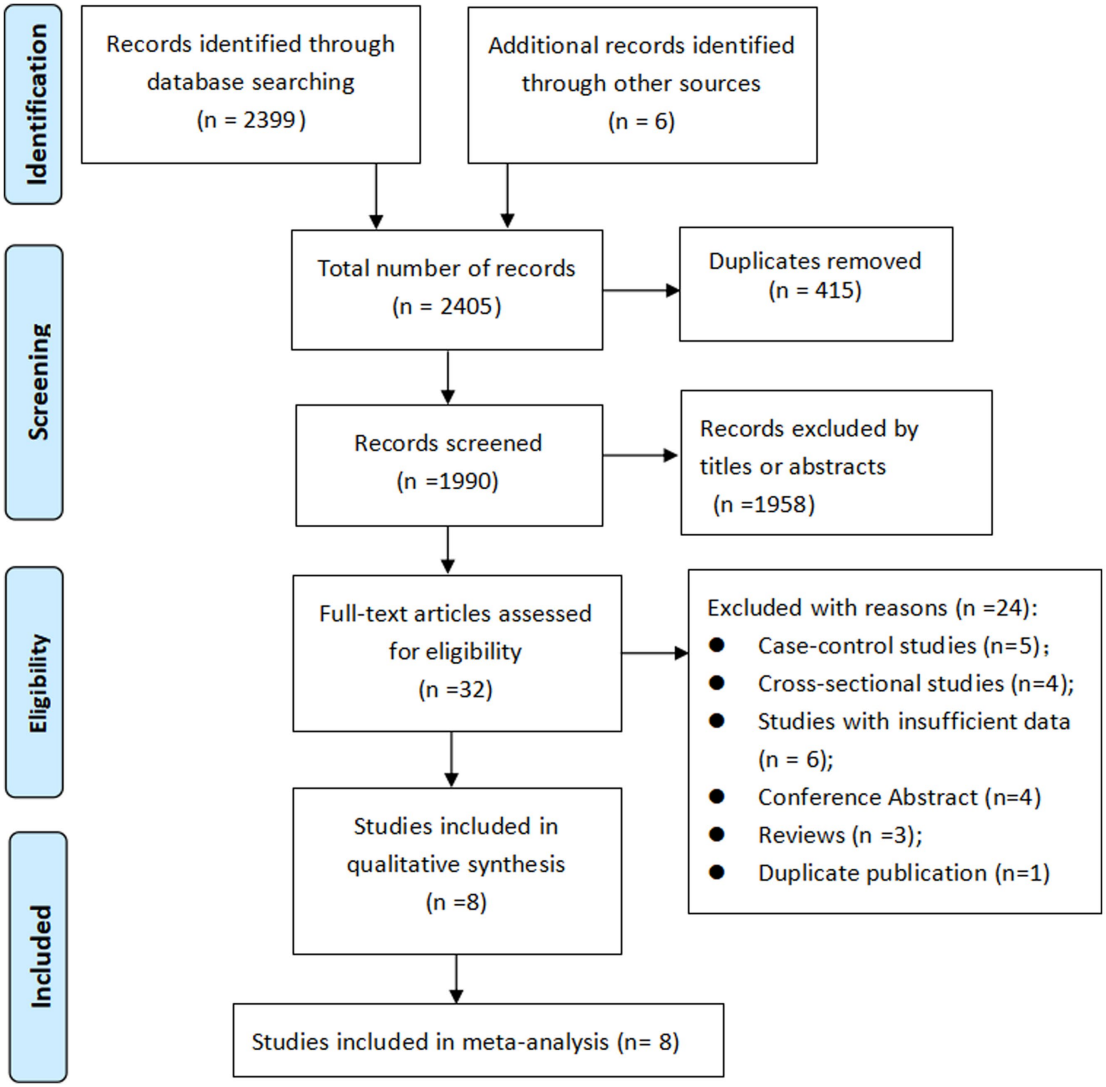
PRISMA flowchart of study selection process.

Initially, 2,405 records were identified, after eliminating duplicate titles, 1990 were retained. Upon review of the titles and abstracts, 1958 records were excluded. The remaining 32 articles were then retrieved for full-text evaluation, leading to the inclusion of 8 cohort studies (20–27) in our meta-analysis. The literature selection process is shown in Figure 1.

### 3.2 Study characteristics

A total of 8 cohort studies involving 1,939,164 participants were included in this meta-analysis. Of the studies included, two were prospective cohorts (22, 25) and six were retrospective cohorts (20, 21, 23, 24, 26, 27). The time frame of the studies included in this meta-analysis ranged from 2006 to 2022. The research population spans Oceania (Australia), Asia (China and South Korea), Europe (Denmark, Sweden, and France), and North America (United States and Canada). The sample size spanned from 1,323 to 1,264,180. With regard to the diagnosis of HCV infection, five studies (20, 21, 23, 24, 27) used laboratory tests and three studies (22, 25, 26) used International Classification of Diseases (ICD) codes. Regarding the diagnosis of CRC, all studies used ICD codes. The median follow-up time of these studies lasted 3.3–8 years. Of the studies evaluated for quality with NOS, one was rated as high-quality (nine stars), seven as medium-quality (seven or eight stars), and none as low-quality. Table 1 provides details of the studies included in the study.

**Table 1:** Basic characteristics of included studies.

References	Publication year	Country	Study design	Study period	Sample size	Diagnosis of HCV infection	Diagnosis of CRC	Follow-up time (median years)	NOS score
Amin et al. (20)	2006	Australia	Retrospective cohort	1990–2002	75, 834	Anti-HCV or HCV RNA	ICD-code	4.9	8
Omland et al. (21)	2010	Denmark	Retrospective cohort	1994–2003	4, 349	Anti-HCV or HCV RNA	ICD-code	3.3	8
Allison et al. (22)	2015	US	Prospective cohort	2006–2010	12,126	ICD-code	ICD-code	5	8
Kamiza et al. (23)	2016	China	Retrospective cohort	2000–2011	44, 150	ICD code	ICD-code	/	8
Liu et al. (24)	2017	Sweden	Retrospective cohort	1990–2010	29, 271	Anti-HCV or HCV RNA	ICD-code	/	7
Allaire et al. (25)	2018	France	Prospective cohort	1980–2012	1, 323	Anti-HCV	ICD-code	5	7
Hong et al. (26)	2020	South Korea	Retrospective cohort	2003–2013	50, 7,931	ICD-code	ICD-code	8	9
Darvishian et al. (27)	2022	Canada	Retrospective cohort	1990–2016	1, 264, 180	Anti-HCV, HCV RNA or genotype test	ICD-code	/	8

HCV, Hepatitis C virus; CRC, colorectal cancer; Anti-HCV, Anti-hepatitis C virus; ICD, International Classification of Diseases; NOS, Newcastle-Ottawa Scale.

### 3.3 Overall meta-analysis of HCV infection and risk of CRC

**Figure 2:**
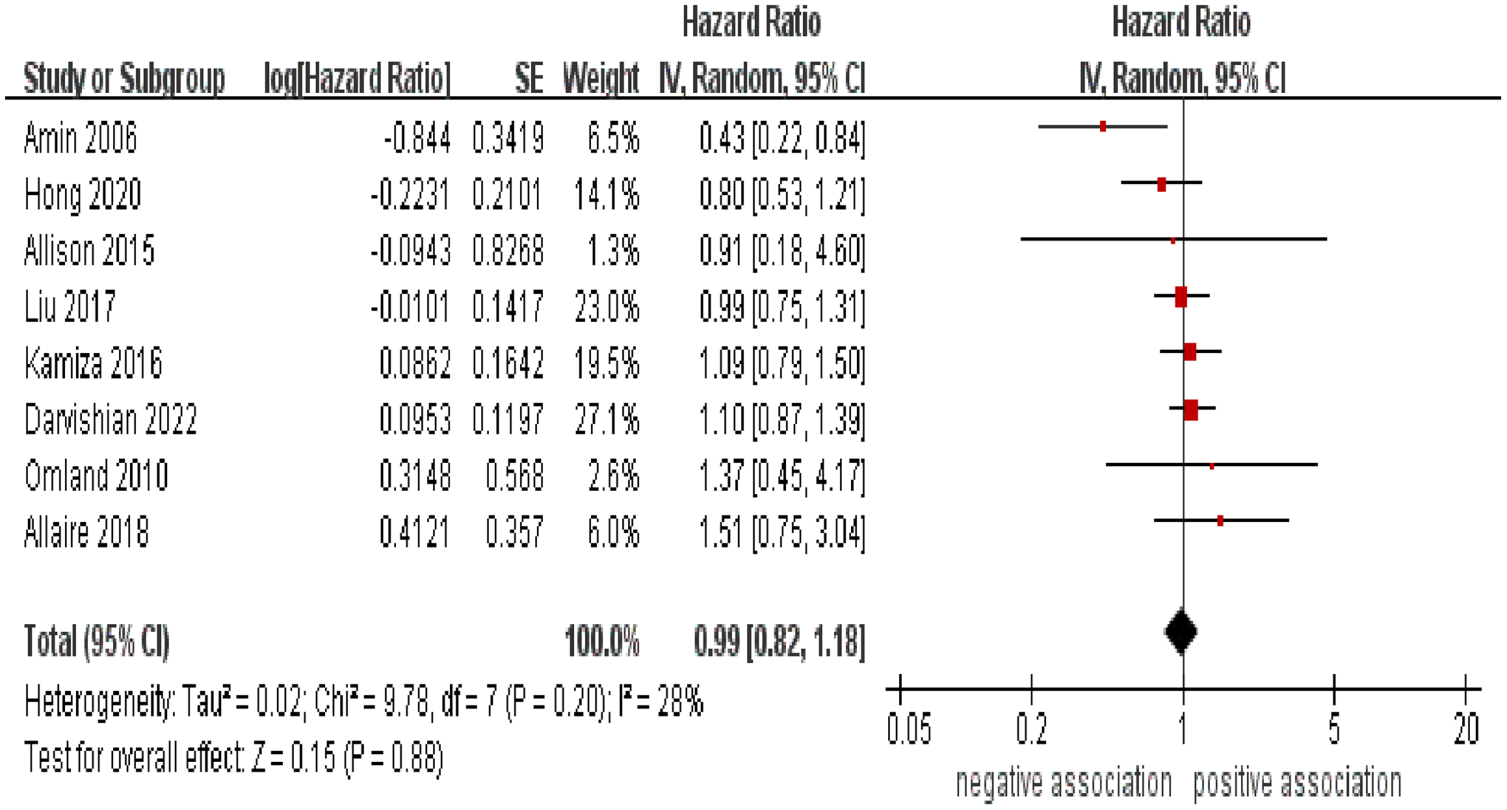
Forest plot of overall meta-analysis of association between HCV infection and risk of CRC.

As shown in Figure 2, the result of the meta-analysis indicated that there was no significant association between HCV infection and the likelihood of developing CRC (pooled HR = 0.99, 95% CI: 0.82–1.88, *p* = 0.88). Low statistical heterogeneity was observed among these included studies (I^2^ = 28%, *p* = 0.20).

### 3.4 Subgroup and sensitivity analyses

**Figure 3:**
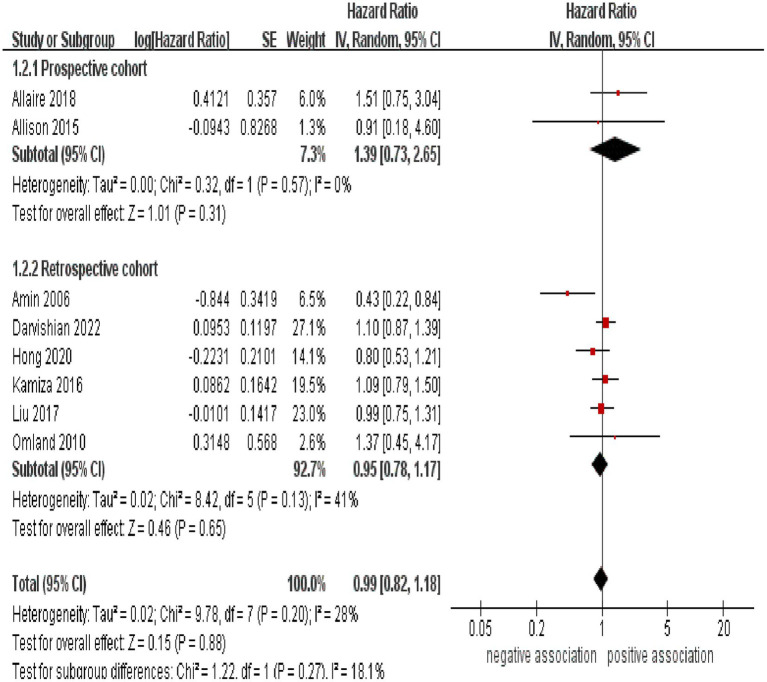
Forest plot of subgroup analysis based on study design.

**Figure 4:**
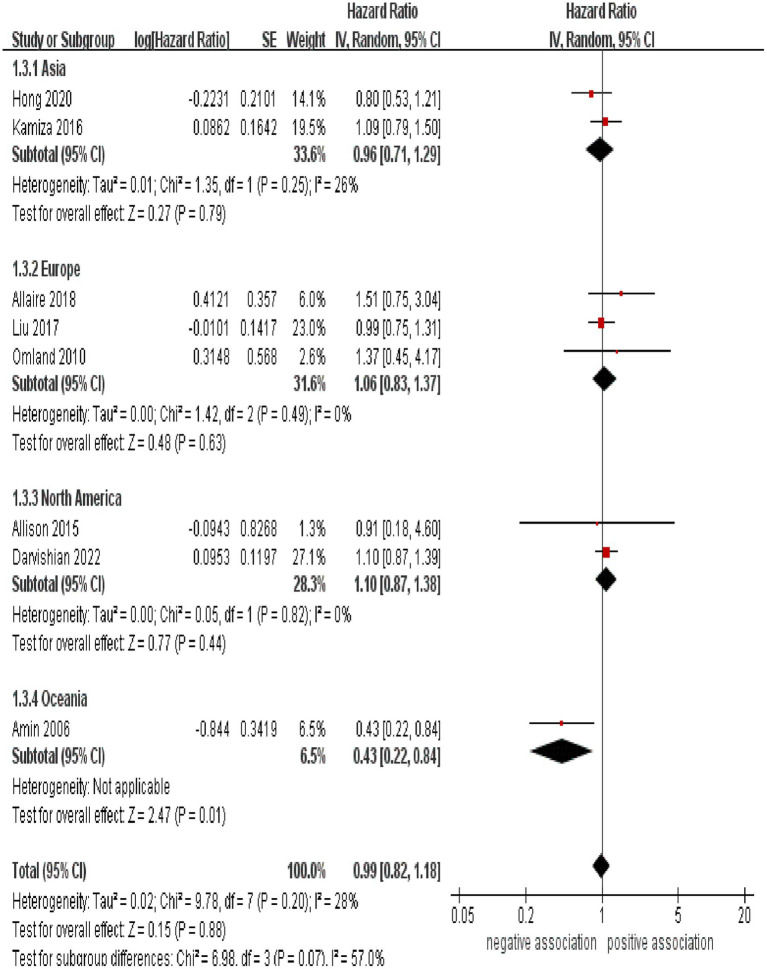
Forest plot of subgroup analysis based on study areas.

**Figure 5:**
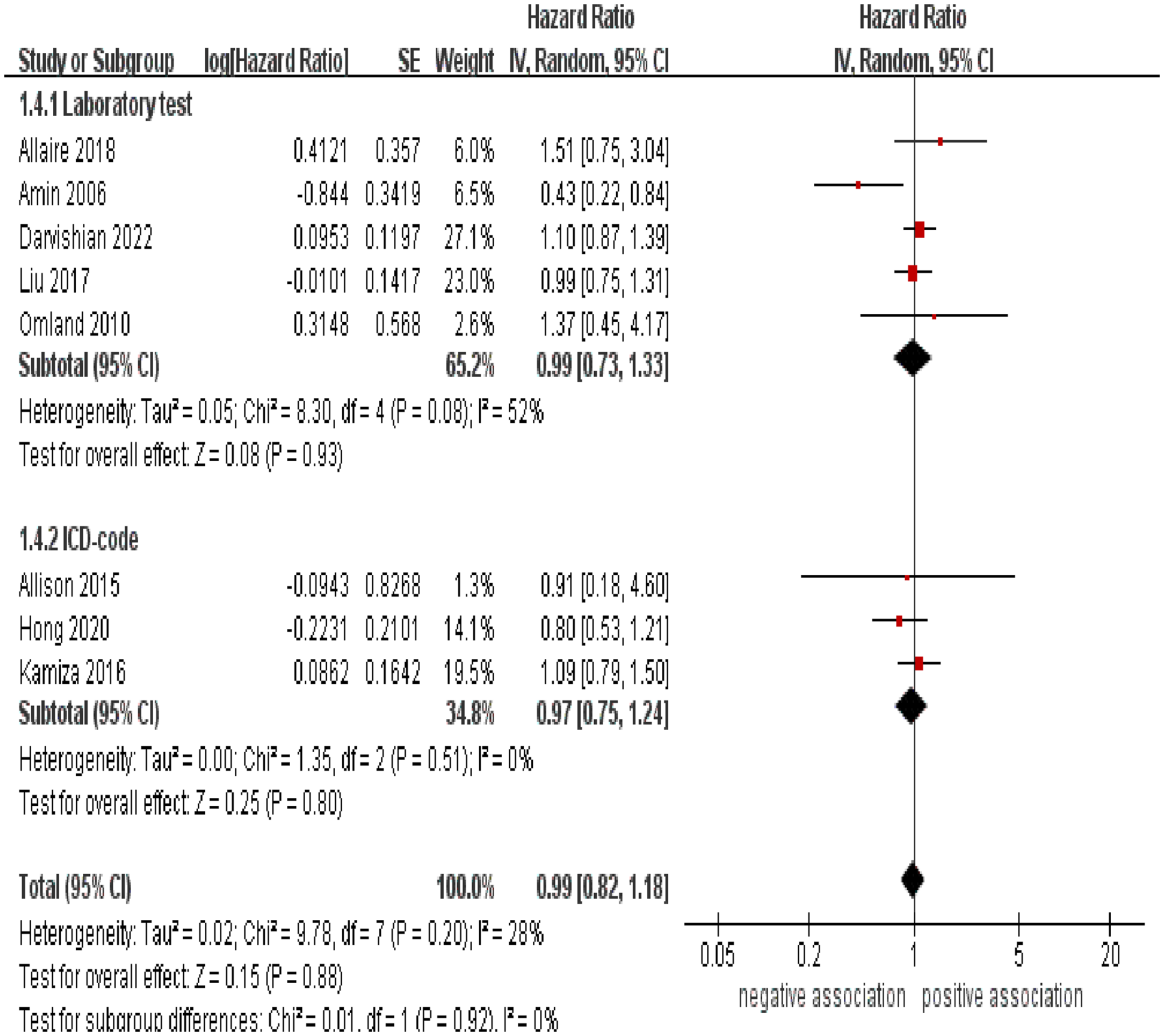
Forest plot of subgroup analysis based on diagnosis of HCV infection.

**Figure 6:**
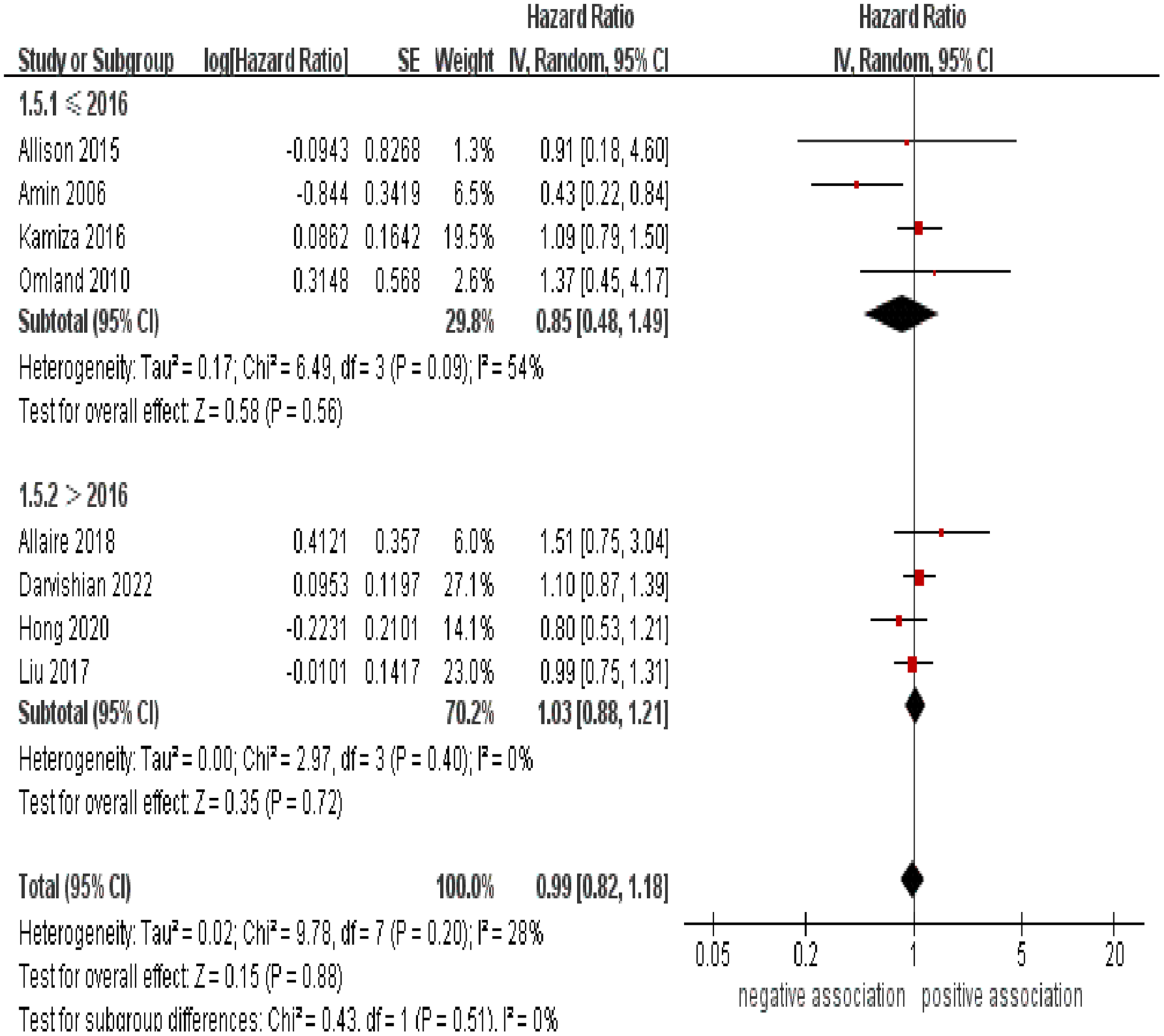
Forest plot of subgroup analysis based on publication year.

Subgroup analyses were performed to assess any potential factors that could influence the overall results and to detect any sources of heterogeneity. The result of subgroup analysis based on study design indicated no significant association between HCV infection and risk of CRC in both prospective (*n* = 2, HR = 1.39, 95% CI:0.73–2.65, *p* = 0.31; I^2^ = 0%) and retrospective cohort studies (*n* = 6, HR = 0.95, 95% CI: 0.78–1.17, *p* = 0.65; I^2^ = 41%) (Figure 3). Analyses of subgroups based on study areas revealed that there was no significant association between HCV infection and CRC risk in Asia (*n* = 2, HR = 0.96, 95% CI: 0.71–1.29, *p* = 0.79; I^2^ = 26%), Europe (*n* = 3, HR = 1.06, 95% CI: 0.83–1.37, *p* = 0.63; I^2^ = 0%), and North America (*n* = 2, HR = 1.10, 95% CI: 0.87–1.38, *p* = 0.44; I^2^ = 0%); however, a negative correlation was found in Oceania (*n* = 1, HR = 0.43, 95% CI: 0.22–0.84, *p* = 0.01) (Figure 4). In a subgroup analysis based on diagnosis of HCV infection, the meta-analysis revealed no significant association between HCV infection and CRC risk when utilizing both laboratory test (*n* = 5, HR = 0.99, 95% CI: 0.73–1.33, *p* = 0.93; I^2^ = 52%) and ICD-code (*n* = 3, HR = 0.97, 95% CI: 0.75–1.24, *p* = 0.80; I^2^ = 0%) (Figure 5). Similar results were observed when conducting subgroup analyses based on publication year (Figure 6). Table 2 displays the results of the subgroup analyses.

**Table 2:** The results of subgroup analyses.

Subgroups	No. of studies	HR (95%CI)	*P*_association_	I^2^ (%)	*P*_heterogeneity_
Study design					
Prospective cohort	2	1.39 (0.73–2.65)	0.31	0	0.57
Retrospective cohort	6	0.95 (0.78–1.17)	0.65	41	0.13
Study areas					
Asia	2	0.96 (0.71–1.29)	0.79	26	0.25
Europe	3	1.06 (0.83–1.37)	0.63	0	0.49
North America	2	1.10 (0.87–1.38)	0.44	0	0.82
Oceania	1	0.43 (0.22–0.84)	0.01	–	–
Diagnosis of HCV infection					
Laboratory test	5	0.99 (0.73–1.33)	0.93	52	0.08
ICD-code	3	0.97 (0.75–1.24)	0.80	0	0.51
Publication year					
≤ 2016	4	0.85 (0.48–1.49)	0.56	54	0.09
> 2016	4	1.03 (0.88–1.21)	0.72	0	0.40
Overall studies	8	0.99 (0.82–1.18)	0.88	28	0.20

HCV, Hepatitis C virus; HR, hazard ratio; CI, confidence interval; ICD, International Classification of Diseases.

Additionally, we conducted a sensitivity analysis by omitting one study at a time and combining the rest. The results were consistent even when any single study was excluded, demonstrating that our results are robust. The sensitivity analyses are summarized in Supplementary Table S2.

### 3.5 Publication bias assessment

As shown in Supplementary Figure S1, the Begg’s funnel plot suggested slight asymmetry, but the Begg’s and Egger’s tests indicated no substantial publication bias in our meta-analysis (*P*_Begg_ = 0.902, *P*_Egger_ = 0.626).

## 4 Discussion

This is the first systematic review and meta-analysis to assess the data from all accessible cohort studies investigating the relationship between chronic HCV infection and the risk of CRC. In this systematic review and meta-analysis of 8 cohort studies involving 1,939,164 participants, no significant association between HCV infection and the risk of developing CRC was observed. Our results were further validated through sensitivity analyses. Similar results were observed when conducting subgroup analyses based on study design, diagnosis of HCV infection, and publication year. Subgroup analysis based on study areas revealed that there was no significant association between HCV infection and CRC risk in Asia, Europe, and North America; however, a negative correlation was found in Oceania (*n* = 1, HR = 0.43, 95% CI: 0.22–0.84, *p* = 0.01).

Previously, Hong et al. (12) conducted a meta-analysis and explored the association between chronic viral hepatitis (hepatitis B virus and HCV infection) and colorectal neoplasia. Their meta-analysis included five studies (two cohort and three case–control studies) and indicated that individuals with HCV infection had a significantly higher risk of colorectal neoplasia than those without HCV infection (OR = 1.88; 95% CI: 1.78–1.97). Our meta-analysis of cohort studies has produced results that contrast with those of prior meta-analyses. In comparison to prior meta-analysis, forgoing case–control study design, which can be more susceptible to bias, we only included cohort studies. By adjusting for covariates, the cohort studies have been able to reduce confounding bias, making the conclusions trustworthy. Furthermore, we included recently published cohort studies and those which had been overlooked in the previous meta-analysis, thus providing more comprehensive and up-to-date evidence of the link between HCV infection and CRC.

Despite our meta-analysis not uncovering a noteworthy association between HCV infection and CRC, a few studies have indicated a possible connection between them. The underlying mechanism, however, is yet to be understood. First, HCV not only infects liver cells, but is also present in the gastrointestinal mucosa, making it an extrahepatic host (28). Studies have revealed that B cells and macrophages in the colon can be infected by HCV as well (29). HCV may directly infect the colon mucosa and cause lesions. Second, it is possible that HCV proteins are implicated in the genesis of CRC (30, 31). HCV core protein has been found to affect the activity of p53, a protein that is vital for DNA repair when it is damaged. Studies have shown that p53 is essential in the progression from colorectal adenomas to CRC in the adenoma-carcinoma sequence (32). HCV core proteins, including E2, NS2, and NS3, have been observed to interact with the MAPK/ERK signaling pathway and some cell cycle proteins, such as cyclin D/CDK4 and cyclin E. This interaction leads to increased cell proliferation, which can result in carcinogenesis (33). By inactivating multiple tumor-suppressor proteins such as p53, p73 and retinoblastoma protein, HCV core protein, NS3 and NS5A protein can lead to cell cycle arrest and apoptosis (30). In addition, NS5A proteins have the ability to obstruct the action of proapoptotic proteins, like caspase-3, Bcl-2, and necrosis factor alpha (TNF-α), which are essential for anti-cancer protection. Moreover, NS5A proteins also can stimulate the generation of antiapoptotic proteins, like Bcl-xl and STAT3, eventually resulting in a weakened response to cancerous growth (30, 33). Third, HCV has the potential to disrupt the immune system due to its ability to infect a variety of immune cells, such as lymphocytes, monocytes, natural killer (NK) cells, and dendritic cells (34–36). HCV core protein, NS2, NS3, and NS4A, which are viral proteins, have been found to reduce gene expression and function of type I interferon and chemokines like CCL5, CXCL8, and CXCL10. These chemokines are vital for NK cell and tumor-infiltrating CD8+ T lymphocyte activation for cancer immunosurveillance and antitumor response (33).

### 4.1 Strengths and limitations

Our meta-analysis has several strengths. Firstly, to our knowledge, this is the first meta-analysis to focus exclusively on cohort studies in relation to this topic, providing up-to-date evidence of the link between HCV infection and CRC. Secondly, we pre-registered our meta-analysis on PROSPERO, using stringent search strategies and criteria for inclusion, in accordance with the PRISMA report statement. The studies we included had a medium to high quality, which make our research results more transparent and dependable. Thirdly, sensitivity analysis was used to validate the stability and reliability of the research results, and publication bias test was conducted to prove the lack of significant publication bias.

While our meta-analysis has these strengths, there are some limitations that should be noted. Firstly, the included studies have varied in their adjustment factors, and some have not fully taken into account modifiable risk factors such as smoking, processed meat, alcohol intake, red meat, intake of vegetables and fruits, physical activity and obesity in CRC, which could affect the estimation of the relationship between HCV infection and CRC risk. Secondly, despite the low statistical heterogeneity (I^2^ = 28%) identified in our meta-analysis, distinctions in the background population and research methods were observed. To better understand these differences, we conducted multiple subgroup analyses and found that the heterogeneity was mainly caused by the various populations in the study areas. Meanwhile, we further conducted sensitivity analysis. We noticed that when the study from Amin et al. (20) in Australia in 2006 was removed, heterogeneity decreased to 0%. This could be due to the fact that the study population was different from other studies, and HCV infection was observed to be negatively correlated with CRC, which contrasts other research results. Furthermore, the study was published earlier, and the colorectal endoscopy examination technology at that time was less advanced, which could have led to an underestimation of the risk between the two diseases. Thirdly, certain studies did not provide a specific duration for the follow-up, and some studies had a shorter follow-up, which may have an impact on the results. Finally, most of the studies did not provide information on the treatment status of HCV, HCV genotype and viral load, cirrhosis and/or non-alcoholic fatty liver, and concomitant HBV or HIV infection. As these factors may affect the association between HCV infection and risk of CRC, further research is necessary.

## 5 Conclusion

Our cohort-based meta-analysis showed insufficient evidence to support the association between HCV infection and an increased risk of CRC. To gain a clearer insight into any association, it would be beneficial to conduct large, well-designed, high-quality prospective cohort studies that consider different ethnic populations and potential confounding factors.

## Data availability statement

The original contributions presented in the study are included in the article/Supplementary material, further inquiries can be directed to the corresponding authors.

## Author contributions

CC: Conceptualization, Formal analysis, Investigation, Methodology, Software, Supervision, Visualization, Writing – original draft, Writing – review & editing. H-MY: Conceptualization, Data curation, Formal analysis, Investigation, Software, Visualization, Writing – original draft. Y-LL: Conceptualization, Data curation, Formal analysis, Methodology, Software, Validation, Visualization, Writing – original draft.
