# Supplementary material for: No association between hepatitis C virus infection and risk of colorectal cancer: a systematic review and meta-analysis of cohort studies
Source: Front Med (Lausanne). 2024 Jun 4;11:1327809. doi: 10.3389/fmed.2024.1327809 (PMC11186414; doi:10.3389/fmed.2024.1327809)

Supplementary online materials

Table S1. **The details of search strategy of three electronic databases**

Table S2. **The results of sensitivity analyses**

Figure S1. **The Begg's funnel plot for overall results**

**Table S1 Search Strategy** (from inception to October 12, 2023)		
**Databases**	**Search strings**	**Results**
**PubMed**	("hepatitis C"[MeSH Terms] OR "hepatitis C"[All Fields] OR "HCV"[All Fields]) AND (("colon"[MeSH Terms] OR "colon"[All Fields]) OR ("rectum"[MeSH Terms] OR "rectum"[All Fields]) OR ("colorectal"[All Fields]) OR ("colons"[All Fields]) OR ("colonic"[All Fields]) OR ("rectal"[All Fields])) AND (("cancer"[All Fields] OR "cancers"[All Fields]) OR ("tumour"[All Fields] OR "tumor"[All Fields]) OR ("tumours"[All Fields] OR"tumors"[All Fields]) OR ("neoplasms"[MeSH Terms] OR "neoplasms"[All Fields] OR "neoplasm"[All Fields]) OR ("carcinoma"[MeSH Terms] OR "carcinoma"[All Fields]) OR ("neoplasia"[MeSH Terms] OR "neoplasia"[All Fields]))	316
**EMBASE**	#1 'hepatitis C'/exp OR 'hepatitis C'	179 645
	#2 'HCV'	112 370
	#3 #1 OR #2	197 829
	#4 'colon'/exp OR colon	497 323
	#5 'rectum'/exp OR rectum	222 583
	#6 'colons' OR 'colonic'	121 290
	#7 'rectal'	203 908
	#8 #4 OR #5 OR #6 OR #7	731 942
	#9 'neoplasm'/exp OR neoplasm	6276 581
	#10 'neoplasms'/exp OR neoplasms	6283 010
	#11 'cancer'/exp OR cancer	6410 792
	#12 'cancers'/exp OR cancers	4790 068
	#13 'tumor'/exp OR tumor	7049 548
	#14 'tumors'/exp OR tumors	6348 756
	#15 'tumour'/exp OR tumour	6330 732
	#16 'tumours'/exp OR tumours	6288 365
	#17 'carcinoma'/exp OR carcinoma	1817 996
	#18 'neoplasia'/exp OR neoplasia	6273 920
	#19 #9 OR #10 OR #11 OR #12 OR #13 OR #14 OR #15 OR #16 OR #17 OR #18	8163 401
	#20 #3 AND #8 AND #19	**1252**

**Web of Science** (Core Collection)	TS=(hepatitis C OR HBV OR HCV) AND TS=(colorectal OR colon OR colons OR colonic OR rectal OR rectum) AND TS=(neoplasm OR neoplasms OR cancer OR cancers OR tumor OR tumors OR tumour OR tumours OR carcinoma OR neoplasia)	**831**

Table S2 Results of sensitivity analyses

**Studies omitted**	**HR (95% CI)**	$P_{association}$	**Heterogeneity**
Amin, 2006	1.04 (0.91-1.20)	0.55	I^2 = 0%, P = 0.77
Omland, 2010	0.97 (0.80-1.18)	0.79	I^2 = 37%, P = 0.15
Allison, 2015	0.98 (0.81-1.20)	0.86	I^2 = 39%, P = 0.13
Kamiza, 2016	0.95 (0.76-1.20)	0.69	I^2 = 37%, P = 0.15
Liu, 2017	0.97 (0.76-1.24)	0.83	I^2 = 39%, P = 0.13
Allaire, 2018	0.96 (0.80-1.16)	0.67	I^2 = 29%, P = 0.21
Hong, 2020	1.02 (0.83-1.25)	0.85	I^2 = 29%, P = 0.21
Darvishian, 2022	0.94 (0.74-1.20)	0.62	I^2 = 33%, P = 0.18

HR, hazard ratio; CI, confidence interval.

Figure S1. The Begg's funnel plot for overall results

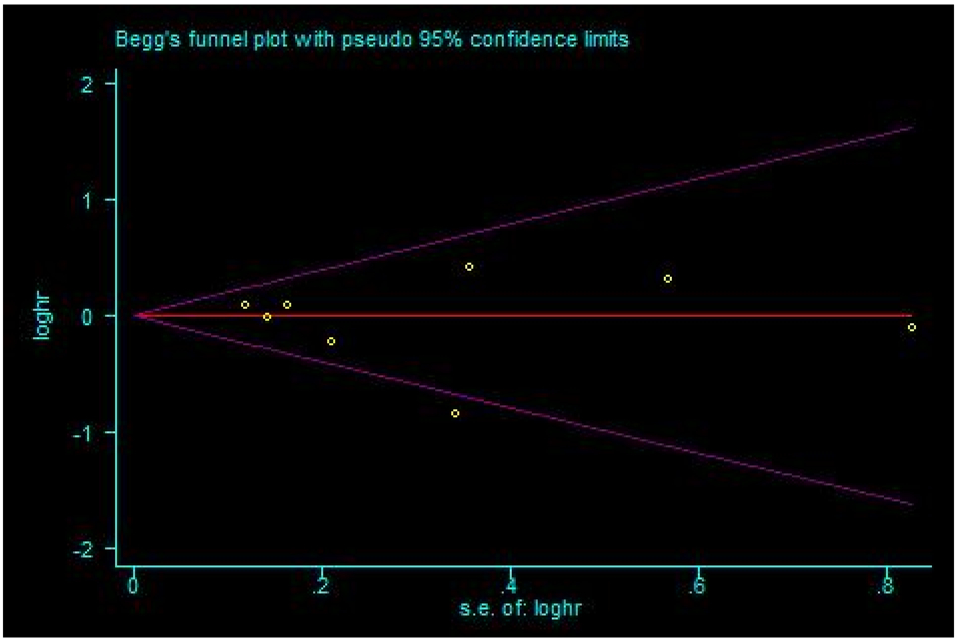

Supplement: Supplementary file 1 [file Data_Sheet_1.PDF]
